# Incidental finding of leukaemia in circulating tumour DNA— the importance of a molecular tumour board

**DOI:** 10.1038/s44276-023-00034-6

**Published:** 2024-02-13

**Authors:** Justin Mencel, Neha Rayarel, Paula Proszek, Paul Carter, Andy Feber, Sanjay Popat, Terri P. McVeigh, Angela George, Alan Dunlop, Katy Hardy, Ian Chau, David Cunningham, Darina Kohoutova, Richard Lee, Sunil Iyengar, Naureen Starling

**Affiliations:** 1 Royal Marsden Hospital, London, UK; 2 Centre of Molecular Pathology, Institute of Cancer Research, London, UK

## Abstract

As the use of liquid biopsies are increasing across multiple indications in cancer medicine, the detection of incidental findings on circulating tumour DNA is of increasing importance. We report the finding of leukaemia detected in a patient who underwent plasma-based circulating tumour DNA next generation screening as part of a screening liquid biopsy study. A *BRAF* V600E mutation detected was deemed pathogenic following discussion at a molecular tumour board, and recommendation of further investigations led to the diagnosis of an occult haematological malignancy. We report the importance of molecular tumour board discussion and recommendations in the identification of incidental, pathogenic findings on circulating tumour DNA.

## Introduction

An 81-year-old man was referred by his general practitioner under the 2-week-wait rule for suspected colorectal cancer (CRC) based on an elevated faecal immunochemical test (FIT) of 12.0 ug/g through an NHS national screening programme in April 2021. At the time, he was experiencing mild symptoms of abdominal discomfort and altered bowel habit, with occasional haematochezia. His medical history included a resected basal cell carcinoma, diverticular disease, and benign prostate hypertrophy. He reported a family history of malignant melanoma. He was seen and assessed by the colorectal surgical team and referred for a colonoscopy to screen for suspected CRC.

At the time of referral, he was considered suitable for the PREVAIL ctDNA Study (NCT04566614). This COVID-19 initiated study was set up to assess the use of circulating tumour DNA (ctDNA) as a tumour agnostic approach, in the earlier and faster diagnosis of patients with suspected cancer, across multiple tumour types including CRC. Patients referred for a screening colonoscopy with an elevated FIT test (≥10 ug/g) were considered eligible, noting the challenges and delays of accessing diagnostic tests during the pandemic.

**Fig. 1:**
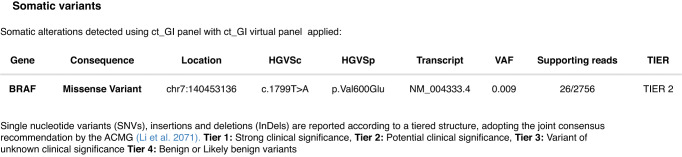
Molecular diagnostic report.

Plasma was collected prior to the colonoscopy, with ctDNA isolated and next generation sequencing (NGS) performed using an in-house multi-gene panel (ct-GI panel) covering 22 genes (Clinical Genomics, Centre for Molecular Pathology, the Royal Marsden, London UK). This panel can detect aberrations commonly seen in CRC, in genes such as *APC*, *KRAS*, *BRAF*, *NRAS*, and *TP53* (see Appendix A). The assay uses unique molecular identifier (UMI)-based background error correction and has >90% sensitivity and specificity to detect single nucleotide variants (SNV) and indels to a variant allelic frequency (VAF) ≥ 0.125% or ≥5 consensus reads. In this pilot study, tumour agnostic (i.e., de novo, plasma-only) variant calling approach was used. Matched panel sequencing data of white blood cell DNA was used to allow discrimination and subtraction of germline and clonal haematopoiesis of indeterminate potential (CHIP) variants. This patient had a pathogenic *BRAF*^*V600E*^ missense somatic variant detected at a VAF of 0.9% (see Fig. 1).

The subsequent screening colonoscopy revealed a single polyp which was fully resected. Histopathological examination confirmed a low-grade dysplastic polyp without evidence of high-grade dysplasia or invasive adenocarcinoma. Tissue based-NGS of the polyp revealed no *BRAF*^*V600E*^ mutation. Subsequent droplet digital PCR (ddPCR) confirmed *BRAF*^*V600E*^ wildtype status in the polyp, consistent with NGS findings.

His case was discussed at the North-West London, Genomic Tumour Advisor Board (GTAB) meeting (molecular tumour board [MTB]), with the aim of determining the origin of the somatic *BRAF* mutation detected and recommendations of further investigations. The MTB consists of genomic and oncology experts who discuss molecular diagnostic reports in the context of clinical history and imaging. The GTAB concluded this plasma-detected *BRAF* mutation was pathogenic, with a high likelihood of being somatic in origin and related to a cancer diagnosis. In the absence of frankly invasive colorectal pathology, the GTAB recommended further investigations to identify an occult malignancy.

**Fig. 2:**
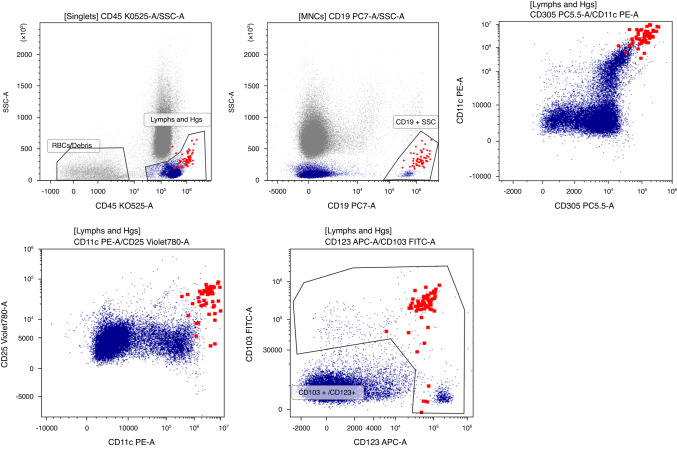
Flow cytometry.

Due to the association of *BRAF* mutations with melanoma and lung cancer, he was subsequently referred to a respiratory physician to screen for a possible lung primary and dermatologist to screen for melanoma. He underwent imaging including a CT neck, thorax, abdomen and pelvis, which revealed a small pancreatic cyst, however, no suspicious mass lesions, including no gastric, small bowel, hepato-biliary, lung masses or skin lesions. FDG-PET scan revealed no FDG avid masses or concerning skin lesions. Subsequent magnetic resonance cholangiopancreatography (MRCP) revealed multiple pancreatic lesions (largest measuring 2.6 cm) without suspicious imaging features. A complete skin examination revealed no skin lesions suggestive of melanoma, however, a basal cell carcinoma resected on the right chest wall was fully excised. A complete blood examination (CBE) revealed mild leukopenia (white cell count 3.5 × 10^9^/L), monocytopenia (0.07 × 10^9^/L) and thrombocytopenia (110 × 10^9^/L). Subsequent peripheral blood flow cytometry showed a clonal B cell population expressing CD25, CD103, CD123 and CD11c, in keeping with a diagnosis of hairy cell leukaemia (HCL) (see Fig. 2). He described no symptoms to suggest leukaemia, including no fatigue, weight loss, night sweats or fevers. ddPCR genotyping of the *BRAF*^*V600E*^ variant (exon 11, 15) was performed on the peripheral blood DNA to confirm the plasma-based NGS sequencing results from the PREVAIL ctDNA study. This revealed no *BRAF* mutation. However, the clonal B cell represented <1% of the total nucleated cells on flow cytometry, and therefore the ddPCR result is likely a false negative given the high likelihood of low-level *BRAF* variants in the blood (sensitivity 97.6% for ddPCR). Given the flow cytometry result and the plasma-based NGS detection of *BRAF* mutation (arising from the malignant B cell nucleus), he was diagnosed with HCL after consultation with a haematologist. At the time, he had no palpable lymphadenopathy or hepato-splenomegaly. His diagnosis was based on an incidental finding on ctDNA analysis through a plasma-only assay. He opted for a watchful waiting approach and remains asymptomatic.

The patient has been monitored for 12 months without symptoms of HCL. His blood counts remain borderline low, however stable with no signs of bone marrow failure or hepatosplenomegaly. Therefore no treatment intervention has been deemed necessary to date. He continues to have erratic bowel symptoms and will undergo a guideline-directed follow-up for polyp surveillance and assessment of the intra-ductal papillary mucinous neoplasm.

## Discussion

*BRAF*^*V600E*^ mutations can be seen across multiple tumour types, most commonly in malignant melanoma (40%), colorectal cancer (10%), lung cancer (<3%) and papillary thyroid cancer (45%) [1–4]. *BRAF*^*V600E*^ mutations are also a hallmark of HCL and can be seen in almost 100% of cases [5]. HCL is a relatively uncommon malignancy, and accounts for only 2% of all lymphoid leukaemias [6]. It is an indolent disease, and usually presents with cytopenias or organomegaly. The diagnosis is often made on peripheral blood morphology and immunophenotyping (B cell expression of CD19, CD20, CD22, CD103, CD123, CD25 and CD11c) in combination with bone marrow aspiration findings. In patients who remain asymptomatic without evidence of significant cytopenia, observation is usually recommended. Treatment is indicated in those with clinically significant cytopenia, organomegaly or symptoms related to HCL with purine analogues forming the mainstay of frontline treatment [7].

Liquid biopsy testing to detect ctDNA is currently being studied and used across multiple indications in cancer, including in screening, early diagnosis, and detection of relapsed disease, molecular profiling of advanced tumours and in longitudinal disease monitoring. Its use in the molecular profiling of advanced tumours to identify potentially targetable variants has established evidence and provides a rapid, minimally invasive alternative to tissue-based molecular diagnostics [8].

The use of ctDNA in cancer screening is of promise with several large-scale population-based screening studies in progress (NCT05611632, NCT05155605, NCT03085888). Although ctDNA analysis in the molecular profiling of advanced tumours is often performed in those with histologically confirmed malignancy, and in monitoring for and early detection of disease relapse, its use in screening asymptomatic patients and in early diagnosis of symptomatic patients in a tumour-agnostic approach using a plasma-only assay is being explored in multiple studies. The risk of detecting false positives in any screening programme is of important relevance, given the potential risk of over-investigation and heightened patient anxiety. When using a plasma-based ctDNA NGS assay, false positives may arise from non-tumour derived DNA, including age-related clonal haematopoiesis (ARCH), CHIP, germline aberrations, and other somatically driven non-tumour derived DNA. CHIP can be seen in 9.5% of individuals aged over 70 years old, with the incidence rising with age [9]. Discrimination of CHIP from variants of somatic origin is possible by using white blood cell (buffy coat) DNA as a matched control [10]. This approach has been validated in a randomised control study of perioperative treatment in patients with gastric cancer (CRITICS Trial) using ctDNA as a predictive biomarker compared with matched buffy coat controls [11]. Germline variants can also be detected by sequencing lymphocyte-derived DNA, although other cell types may be more appropriate in patients with haematological malignancies. Circulating somatic mutations can also be seen in a small proportion of non-malignant related conditions such as in inflammatory diseases, and can arise from benign tissue at low allelic frequencies [12]. Recently, the GRAIL Pathfinder study assessed the use of ctDNA methylation (using the Galleri test) to detect a cancer signal across multiple tumour types. Although the false positive rate was very low (0.8%), there is a potential to detect non-tumour derived variants in the plasma of asymptomatic patients when using ctDNA as a screening tool [13].

With the increase of ctDNA based research studies and screening programmes, it is important to reflect on the many challenges of this technology. The detection of ctDNA in an asymptomatic patient with normal imaging, in particular, requires a focused diagnostic approach. Targeted investigations should be stratified depending on the aberration detected and the patient symptoms. This could help to avoid unnecessary patient anxiety, and reduce the risk of over-investigation or a surge in additional burden on diagnostic services, particularly in the setting of large, population based ctDNA screening trials. It is increasingly recognised that such patients can benefit from discussion in molecular tumour boards (MTB) involving genomic and clinical experts, to interpret reported variants (particularly when using a plasma-only assay without prior knowledge of the tumour genome). The MTB’s role in the interpretation of these ctDNA aberrations can give clinical context and provide recommendations for further targeted investigations. Given the increased uptake of plasma-only assays across cancer medicine, it is imperative that we then also develop robust genomic and diagnostic infrastructure, such as these MTBs, and downstream services such as rapid diagnostic centres that will help to enable better, and earlier cancer diagnosis and treatment.

We report a case of an otherwise asymptomatic patient who was initially investigated for CRC, through plasma-only analysis of ctDNA a *BRAF* mutation was detected, and subsequent MTB discussion led to the diagnosis of hairy cell leukaemia.

## Supplementary information

Supplementary Data
