# Supplementary material for: Incidental finding of leukaemia in circulating tumour DNA— the importance of a molecular tumour board
Source: BJC Rep. 2024 Feb 13;2:12. doi: 10.1038/s44276-023-00034-6 (PMC11524082; doi:10.1038/s44276-023-00034-6)
Supplement: Supplementary file 1 — Supplementary Data [file 44276_2023_34_MOESM1_ESM.docx]

Supplementary Data

**Figures**

Fig 1. Molecular diagnostic report


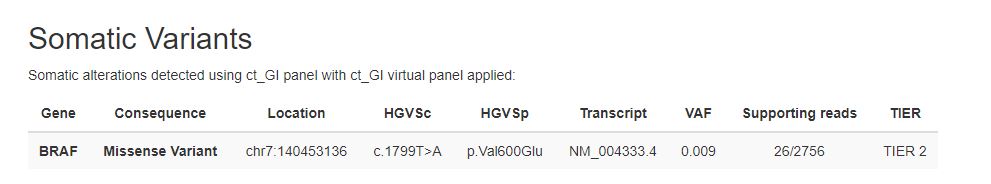


Fig 2. Flow cytometry

A small population of B cells (highlighted in red) show a distinct immunophenotype in keeping with hairy cell leukaemia, positive for CD19, CD123, CD103, CD11C, CD25 and CD305 with increased side scatter.

**Patient consenting**

Consent to publish this case including the images was given by the patient in accordance with local good clinical practice guidance.

**Appendices**

Appendix A- ct-GI Panel Coverage

| **Gene** | **Location** |
| --- | --- |
| *ACVR2A* | Exon 10 |
| *AKT1* | Exon 3 |
| *APC* | Exons 1-16 |
| *ARID1A* | Exons 1-20 |
| *ATM* | Exons 1-63 |
| *BRAF* | Exons 11,14-16 |
| *CTNNB1* | Exons 3,7-8 |
| *DOCK2* | Exons 1-52 |
| *EGFR* | Exons 18-21 |
| *FBXW7* | Exons 1a,1b,1c-12 |
| *KRAS* | Exons 2-3 |
| *NOTCH1* | Exons 1-34 |
| *NRAS* | Exons 2-3 |
| *PIK3CA* | Exons 2-21 |
| *PTEN* | Exons 5,7 |
| *QC* | Exons 1-20 |
| *RET* | Exons 10-11,15-16 |
| *RNF43* | Exons 2-10 |
| *SMAD2* | Exons 2-11 |
| *SMAD4* | Exons 2-12 |
| *TCF7L2* | Exons 1-14 |
| *TGFBR2* | Exon 7 |
| *TP53* | Exons 4-10,10a-11 |
